# The impact of coalition characteristics on outcomes in community-based initiatives targeting the social determinants of health: a systematic review

**DOI:** 10.1186/s12889-022-13678-9

**Published:** 2022-07-15

**Authors:** Phoebe Nagorcka-Smith, Kristy A. Bolton, Jennifer Dam, Melanie Nichols, Laura Alston, Michael Johnstone, Steven Allender

**Affiliations:** 1 Deakin University, Global Obesity Centre (GLOBE), Institute for Health Transformation, School of Health and Social Development, 1 Gheringhap Street, Geelong, VIC 3220 Australia; 2 Deakin University, Institute for Physical Activity and Nutrition, School of Exercise and Nutrition Sciences, 1 Gheringhap Street, Geelong, VIC 3220 Australia; 3 Monash University, Monash Sustainable Development Institute, 8 Scenic Boulevard, Clayton, VIC 3800 Australia; 4 Deakin University, Deakin Rural Health, Faculty of Health, Princes Hwy, Warrnambool, VIC 3280 Australia; 5 Research Unit, Colac Area Health, 2-28 Connor St, Colac, Victoria 3250 Australia; 6 Deakin University, Institute for Intelligent Systems Research and Innovation, 75 Pigdons Road, Waurn Ponds, VIC 3216 Australia

**Keywords:** Collaboration, Coalitions, Coalition Functioning, Coalition Impact, Community-Based Prevention, Health Promotion

## Abstract

**Background:**

Coalitions are a popular mechanism for delivering community-based health promotion. The aim of this systematic review was to synthesize research that has quantitatively analyzed the association between coalition characteristics and outcomes in community-based initiatives targeting the social determinants of health. Coalition characteristics described elements of their structure or functioning, and outcomes referred to both proximal and distal community changes.

**Methods:**

Authors searched six electronic databases to identify peer reviewed, published studies that analyzed the relationship between coalition characteristics and outcomes in community-based initiatives between 1980 and 2021. Studies were included if they were published in English and quantitatively analyzed the link between coalition characteristics and outcomes. Included studies were assessed for quality using the Joanna Briggs Institute analytical cross-sectional studies assessment tool.

**Results:**

The search returned 10,030 unique records. After screening, 26 studies were included from six countries. Initiatives targeted drug use, health equity, nutrition, physical activity, child and youth development, crime, domestic violence, and neighbourhood improvement. Community outcomes measured included perceived effectiveness (*n*=10), policy, systems or environment change (*n*=9), and community readiness or capacity (*n*=7). Analyses included regression or correlation analysis (*n*=16) and structural equation or pathway modelling (*n*=10). Studies varied in quality, with a lack of data collection tool validation presenting the most prominent limitation to study quality. Statistically significant associations were noted between community outcomes and wide range of coalition characteristics, including community context, resourcing, coalition structure, member characteristics, engagement, satisfaction, group facilitation, communication, group dynamics, relationships, community partnership, and health promotion planning and implementation.

**Conclusion:**

Existing literature demonstrates that coalition characteristics, including best practice health promotion planning and evaluation, influence community outcomes. The field of coalition research would benefit from more consistent description and measurement of coalition characteristics and outcomes, and efforts to evaluate coalitions in a wider range of countries around the world. Further research using empirical community outcome indicators, and methods that consider the interrelationship of variables, is warranted.

**Trial registration:**

A protocol for this review was registered with PROSPERO (CRD42020205988).

**Supplementary Information:**

The online version contains supplementary material available at 10.1186/s12889-022-13678-9.

## Contributions to the literature

- This systematic review is the first known to focus exclusively on international research quantitatively analyzing the associations between coalition characteristics and outcomes
- In a field with many competing theoretical frameworks, the review outlines which relationships between coalition characteristics and outcomes have empirical evidence behind them, and which do not
- The review provides a basis for health promotion coalitions to structure their development and work upon, globally

## Introduction

Health promotion aims to address the health and social conditions that drive health outcomes [1], known as the social determinants of health (SDOH). The SDOH encompass the economic, environmental and social conditions that influence the differences in health status experienced by groups and individuals within a population, and include: the social gradient, early life, work, unemployment, social support, addiction, food, education, health services, colonialism, gender, and disability [2, 3].

Internationally, there is agreement that health promotion is done most effectively when interventions are place-based [4]. That is, focusing on structural determinants above individual behavior change, understanding multiple drivers of the health outcome(s), and designed and implemented in partnership with the local community [1]. Such initiatives require collaborative work, or coalitions, to plan and implement strategies across the community or target setting [5, 6]. A health promotion coalition is a group of individuals, organisations, community groups, or other bodies, who undertake joint work including planning, resourcing and implementation, in order to achieve an agreed goal [5, 7, 8]. Coalition approaches, such as the Community Coalition Action Theory (CCAT) [9] or Collective Impact [5], underpin large health promotion initiatives such as Healthy Cities [8], Communities That Care [10], the Whole of Systems Trial of Prevention Strategies for Childhood Obesity (WHOSTOPS) [11], and Healthy Together Victoria [12]. Coalition working has also been mandated through government policy and funding schemes in places such as the United States of America [13], and the United Kingdom [14].

Coalition building has been approached theoretically from perspectives as diverse as business consulting, human rights, and collectivism [5, 6, 15, 16]. Each provides differing perspectives; the business consulting approach prioritises efficiency, and frames collaborative practice as adding value to health promotion work in terms of resourcing, reach, or scope of change [5]; a human rights approach prioritises power, and frames coalitions as a mechanism for people who are typically unheard to contribute to decisions that impact themselves and their communities [15, 16];meanwhile the collectivist approach prioritises partnership ‘synergy’, which describes a belief that collaborative culture produces better resourcing, decision making and impact that would not be possible outside of a coalition approach [6].

Much like the health and social issues they are formed to address, coalitions are complex. Collaborative work commonly brings together people from multiple sectors, resourcing levels, degrees of individual and organisational power, lived experiences, priorities and perspectives [5, 15, 16]. In an attempt to evaluate and optimise the work of coalitions, a number of studies have emerged that aimed to define and measure characteristics critical to their success [7, 9, 17, 18]. Some researchers have translated research from other disciplines, such as management practice [17], to explore which coalition characteristics are likely to influence community outcomes. Others have looked at qualitative reflections from practitioners involved with coalitions and attempted to synthesize them [19], while yet others have turned to their own direct health promotion practice for inspiration [20, 21].

There appears to be a broad range of potential measures in evaluating the impact of coalitions. For example, characteristics can refer to both structural and functional elements of coalitions, such as resourcing, governance and management, member characteristics, member engagement, communication, relationships, group dynamics, community partnership, and the adoption of best practice health promotion planning, implementation and evaluation [6, 16, 21, 22]. Previous attempts to define the characteristics of coalitions assume coalitions are effective implementation mechanisms [7], and that their function influences their outcomes [16]. These assumptions have not been well evaluated, and the most efficient and effective ways of working for coalitions to achieve improvements in the social determinants of health are not well understood.

The aim of this systematic review was to synthesize empirical research that quantitatively analyzed the association between coalition characteristics and outcomes in community-based initiatives targeting the SDOH.

## Methods

### Inclusion criteria

Researchers constructed the search strategy using PRISMA [23] and PROSPERO [24] guidelines for systematic searching, and registered it with PROSPERO [25]. Studies were included that met the following criteria:

- described community-based primary prevention initiative(s) targeting at least one social determinant of health
- in free living human populations
- utilised a coalition model
- conducted a quantitative analysis of the association between coalition characteristics and community outcomes
- peer-reviewed, original research
- published from 1980 to May 2021
- English language

The search was not restricted by study design, however authors excluded studies if they did not quantitatively analyze the relationship between coalition characteristics and outcomes. Coalition characteristics were defined as elements of coalition structure or functioning, and coalition outcomes referred included both proximal (e.g. readiness to change, social capital) and distal (e.g. health outcomes, policy change) community-level changes. Studies were excluded if they reported on individual behavior change rather than community-level prevention, only analyzed associations between coalition characteristics (i.e. only process indicators), or only exhibited community participation below the level of ‘partnership’ on Arnstein’s Ladder of Citizen Participation [26]. Reviews and meta-analyses were excluded, and their references examined for relevant studies.

### Search strategy

Researchers conducted the search in May 2021 using six electronic databases; Medline, Embase, Global Health, Informit Health Collection, SocINDEX, and Cochrane Library. Search terms were based around the four key concepts of ‘collaboration’, ‘community-based initiatives’, ‘prevention of health and social issues’, and ‘evaluation’ (see Additional file 1).

One author (PNS) carried out all database searches, citation management, and uploading to Covidence systematic review software (Veritas Health Innovation, Melbourne, Australia). Covidence removed many duplicates automatically, with additional duplicates removed through the screening process. Two researchers (PNS and LA or JD or KB or MJ) independently screened all papers based on pre-determined eligibility criteria, first by title and abstract, and then by full text. Conflicting assessments were discussed and resolved by consensus between PNS and JD.

### Data extraction and analysis

One author (PNS) extracted all data using a data schema (Additional file 2), with a second author (LA) independently cross-checking a 10% sample for accuracy. The quality of each study was assessed by PNS using the Joanna Briggs Institute Checklist for Analytical Cross Sectional Studies, with JD cross-checking 10% of articles for accuracy [27]. This tool was used to evaluate the appropriateness of the study design, data collection instruments, data analysis, and study reporting. The checklist allows each study to be given an objective rating (yes, no, unclear) on eight domains, with a score of 1 being given for each ‘yes’ rating, a score of 0 for each ‘no’ or ‘unclear’ rating, and a maximum score of 8.

Researchers included associations between coalition structure or function and coalition outcomes in the analysis if they were statistically significant. Researchers adhered to each authors’ own definition of both outcomes and statistical significance, excluding results described as ‘approaching significance’ or similar. The Community Coalition Action Theory (CCAT) framework informed thematic groupings, under headings such as ‘coalition resources’, ‘member engagement and satisfaction’ and ‘planning and implementation’.

## Results

**Fig. 1:**
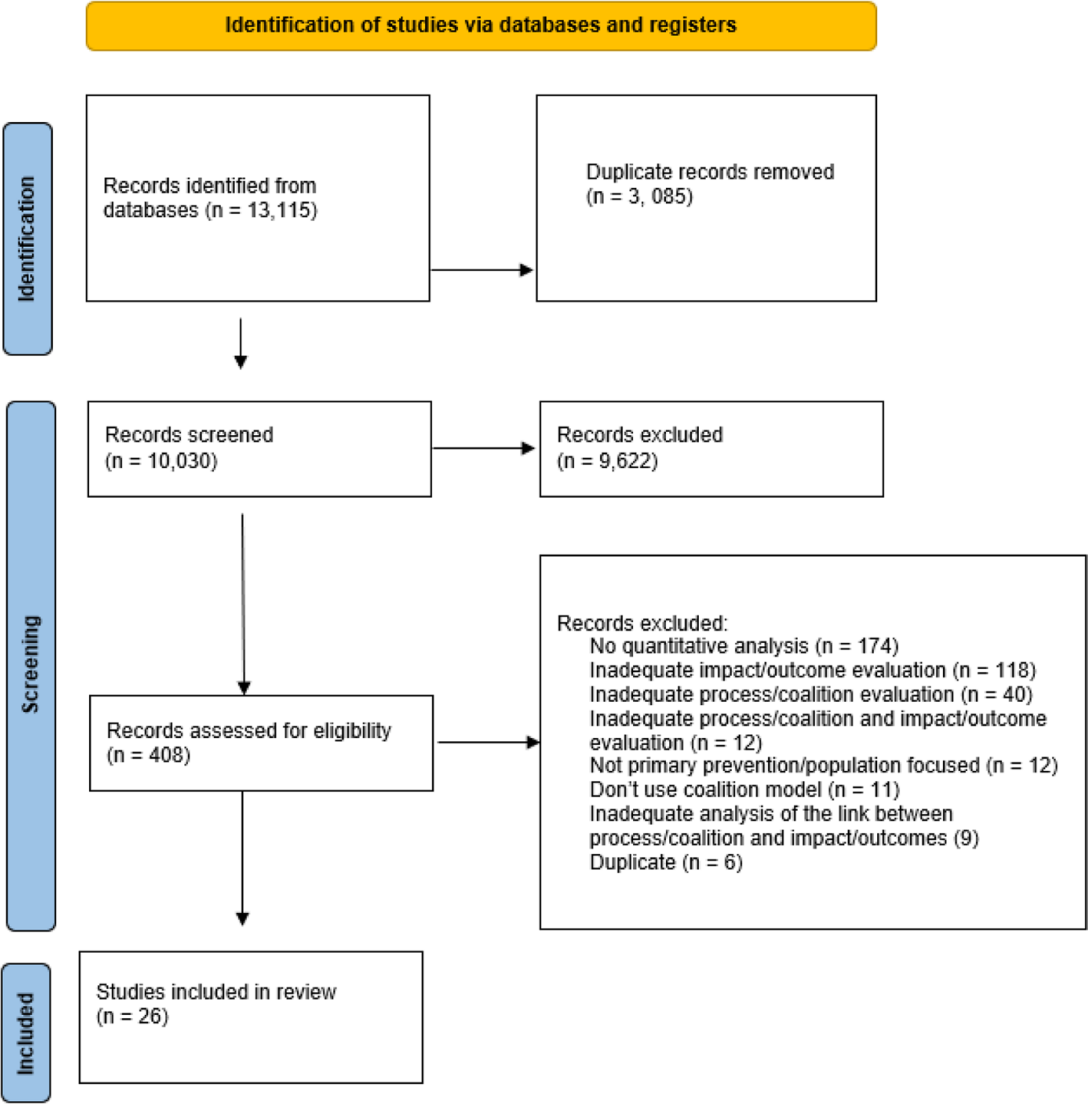
PRISMA diagram of systematic screening. Adapted from: Page MJ, McKenzie JE, Bossuyt PM, Boutron I, Hoffmann TC, Mulrow CD, et al. The PRISMA 2020 statement: an updated guideline for reporting systematic reviews. BMJ 2021;372:n71. doi: 10.1136/bmj.n71

The search retrieved 13,115 articles in total. Thirty-four reviews were excluded and hand searching of the reference lists of these reviews yielded one further paper. A total of 26 studies met the inclusion criteria ([Insert Fig. 1 here]

### Study characteristics

Researchers extracted data from 26 studies, published between 1996 and 2019. Studies were unevenly distributed globally, with the majority of studies (*n*=20) conducted in the USA (Table 1). The most common study design was cross sectional (*n*=12) [28–39], and seven studies did not specify a study design [13, 40–45]. Of those, based on the study description, it is likely that four (*n*=4) [13, 40, 41, 45] were cohort studies, two (*n*=2) [42, 43] were quasi-experimental and one (*n*=1) [44] was cross sectional.

**Table 1:** Selected study characteristics

Summary characteristics	*n*^a^	*%*^b^
Location		
USA	20	77
Israel	2	8
Mexico	1	4
United Kingdom	1	4
Italy	1	4
Malaysia	1	4
Participants		
People (range)	18 - >19,633	
Coalitions (range)	2 - 551	
Study design		
Cross sectional	12	46
Quasi-experimental	2	8
Mixed methods	2	8
Randomised controlled trial	2	8
Case study	1	4
Not specified	7	27
Theoretical framework		
Collaborative capacity (various)	9	35
Community Based Participatory Research	3	12
Organisational development	3	12
Community readiness to change	3	12
Health promotion framework	3	12
Empowerment theory	2	8
Social network theory	2	8
Other	3	12
Condition (SDOH) data collection tool		
Survey with self-reported ratings	2	8
Community survey	2	8
Case studies	1	4
No data collected	21	81
Exposure (coalition characteristics) data collection tool		
Survey with self-reported ratings	20	77
Survey with researcher ratings	1	4
Interview	3	12
Survey/interview and document scan	2	8
Outcome (community change) data collection tool(s)		
Coalition survey	22	85
Interview	6	23
Document scan	4	15
Observational data collection	2	8
Outcome (community change) indicators		
Perceived effectiveness	10	38
Policy, systems, environment change	9	35
Community readiness / capacity	7	27
Social capital	6	23
Partner capacity	4	15
Interagency coordination	4	15
Empowerment	4	15
Health condition / risk factor prevalence	3	12
Analysis type		
Correlation / regression	16	62
Statistical or pathway modelling	10	38
Other	3	12
Total	**26**	**100**

^**a**^Some studies contain multiple tools, indicators or analyses, so totals in each section may not equal* n=*26 or 100%

^**b**^Rounded to the nearest whole number

### Coalitions

All studies collected data from more than one coalition (range: 2 to 551 coalitions, 18 to >19,663 participating coalition members). Seven studies did not provide a total number of participants: [13, 40, 41, 43, 46–48] three provided the number participating in different data collection waves noting that there was an unclear cross-over in respondents [41, 43, 48], three provided the number of coalitions or organisations only [13, 40, 47], and one study did not provide any descriptive data about their participants, including number [46].

Fourteen studies were state-wide or regional efforts comprised of multiple communities using the same implementation framework, such as the Strategic Prevention Framework State Incentive Grant [SPF-SIG], Healthy Cities, or Communities That Care [13, 28–31, 33, 34, 36, 37, 40, 41, 46, 48, 49]. A smaller number of studies investigated coalitions with structural or contextual differences [42, 50, 51], or that were linked through a funding program or community of practice [32, 35, 38, 43, 46, 52]. Ten studies included coalitions that were formed in response to a funding opportunity and/or government policy [13, 30, 31, 34, 36, 37, 41, 43, 45, 46], four included coalitions formed in response to a research project [33, 48–50], three included coalitions that were explicitly grassroots [47, 50, 51], and ten studies included coalitions with unclear origins [28, 29, 32, 35, 38–40, 42, 44, 52]. Compared to nation-wide projects, the three grassroots coalitions tended to focus on discrete programs or problem solving, did not have guiding theoretical frameworks, and used the coalition model to increase their access to decision makers and funding bodies [47, 50, 51].

Eight coalitions targeted multiple health determinants, including neighbourhood improvement, substance use, educational attainment, violence, nutrition, physical activity, unemployment, and housing [28, 29, 31, 33, 34, 41, 48, 51]. Coalitions with a single focus targeted alcohol and other drug use (*n*=7) [36, 38, 45, 46, 49, 50, 52], family violence (*n*=2) [39, 47], health equity (*n*=2) [30, 37], youth empowerment (*n*=1) [43], early childhood development (*n*=1) [40], food environments (*n*=1) [44], and environmental issues (*n*=1) [42]. Two studies did not specify the focus of the coalitions [35, 40]. Table 2 provides a summary of each of the included studies.

**Table 2:** Summary results of included studies

Author	Year	Location	Study design	Coalition name	Formation	Theoretical framework	Participants	Exposure (coalition characteristics) measurement tool	Outcome indicators
Allen et al. [39]	2012	USA: Mid-west	Analytical cross-sectional	Family Violence Coordinating Councils (FVCC)	Unclear	Own, including collaborative capacity, social capital, and empowerment concepts	671 participants 21 coalitions	Self-rated survey	Social capital, member empowerment, community readiness/capacity, institutionalised change
Anderson-Carpenter et al. [46]	2017	USA: Kansas	Pre-test, post-test	Kansas Strategic Prevention Framework State Incentive Grant (SPF-SIG)	Policy/funding response	Tri-Ethnic Center for Prevention Research Communtiy Readiness Model	7 coalitions	Self-rated survey Document scan	Community readiness/capacity
Brown et al. [28]	2017	Mexico	Analytical cross-sectional	Red de Coaliciones Comunitarias de Mexico	Unclear	Collaborative capacity (Foster-Fishman 2001) Work group (Hackman 1987)	211 participants 17 coalitions	Self-rated survey	Community readiness/capacity, community improvement attributable to the coalition, sustainability planning
Calancie et al. [44]	2018	USA, Canada, Native American Tribes and First Nations	Not specified *(analytical cross sectional)*	Food Policy Councils (FPCs)	Various	FPC Framework (Allen et al. 2012)	354 participants 95 coalitions	Self-rated survey	Social capital, perceived effectiveness
Cicognani et al. [29]	2019	Italy: Emilia-Romagna region	Retrospective, cross-sectional	Guadagnare Salute in contesti di Comunita [Gaining health in community contexts]	Unclear	Sense of community (Nowell & Boyd 2010, 2014) Empowerment (Perkins & Zimmerman 1995; Powell & Peterson 2014)	238 participants 6 coalitions	Self-rated survey	Empowerment, perceived efficacy, community readiness/capacity
Crowley et al. [45]	2000	USA	Not specified *(retrospective cohort)*	Community Coalition Program	Policy/funding response	Community-based prevention	Participants not specified >123 coalitions	Self-rated survey	Community readiness/capacity, risk and protective factor prevalence (knowledge, behaviour, attitudes, environment/systems)
Donchin et al. [30]	2006	Israel	Analytical cross-sectional	Healthy Cities Israel	Policy/funding response	Health for All & Agenda 21	18 participants 18 coalitions	Survey with researcher ratings	Policy and political support, policy change, best practice health promotion activities, environmental protection actions
Drach-Zahavy et al. [31]	2006	Israel	Analytical cross-sectional	Healthy Community Centers	Policy/funding response	Ottawa Charter for Health Promotion	37 participants 37 coalitions	Interview	Perceived effectiveness
Duran et al. [32]	2019	USA	Analytical cross-sectional	not specified & various	Unclear	Community-Based Participatory Research	450 participants 164 coalitions	Interview	Partnership synergy, partner and agency capacity, equal power, partnership sustainability, community health and transformation
Emshoff et al. [47]	2007	USA: Georgia	Not specified *(cohort)*	Family Connection	Unclear	Not specified	participants not specified 157 coalitions	Self-rated survey	Shared and inclusive decision making, financial resources, accessible services
Feinberg et al. [33]	2004	USA: Pennsylvania	Analytical cross-sectional	Communities That Care	Research project	Own model based on community readiness and organisational development frameworks	203 participants 21 coalitions	Interview	Perceived effectiveness
Flewelling et al. [12]	2016	USA: 26 states	Not specified *(cohort)*	SPF SIG	Policy/funding response	CSAP Strategic Prevention Framework	318 coalitions (process) 129 coalitions (outcome)	Self-rated survey	Alcohol consumption
Kegler et al. [34]	2012	USA: California	Analytical cross-sectional	California Healthy Cities and Communities	Policy/funding response	Community Coalition Action Theory	231 participants 19 coalitions	Self-rated survey	Community capacity, social capital, sense of community
Lawless et al. [41]	2010	UK: England	Not specified *(cohort)*	New Deal Communities	Policy/funding response	Government policy focusing on evidence, strategy, and locality	19,574 (wave 1) 19,633 (wave 2) 15,792 (wave 3) participants (outcome data) 39 participants (coalition data) 39 coalitions	Self-rated survey	Spend, outputs, project-level reviews, improved schools, police and health facilities
Mansergh et al. [50]	1996	USA: Indianapolis & Pasadena	Case study	Day One Coordinating Council, I-STAR Community Action Council	Research project Grassroots	Butterfoss et al. (1993) collaboration model	100 participants 2 coalitions	Self-rated survey	Coalition efficiency, outcome efficiency (AOD use), interagency coordination
Nowell et al. [47]	2011	USA: Mid-west	Mixed methods	Not specified	Grassroots	Authors’ own	614 organisations 51 coalitions	Self-rated survey	Partner organisation capacity
Oetzel et al. [35]	2018	USA	Analytical cross-sectional & case study	Research for Improved Health study	Unclear	Community-Based Participatory Research	650 participants 200 coalitions	Interview Self-rated survey Document scan	Agency capacity building, personal capacity building, sustainability of the work
Powell et al. [36]	2014	USA	Cross sectional	SPF SIG	Policy/funding response	Psychological Empowerment and Organisational Efforts	138 participants 11 coalitions	Self-rated survey	Psychological empowerment, sense of community, perceived effectiveness
Ramanadhan et al. [37]	2012	USA	Analytical cross-sectional	Massachusetts Community Network for Cancer Education, Research, and Training (MassCONECT)	Policy/funding response	Community-Based Participatory Research	38 participants 3 coalitions	Self-rated survey	Community activities, grants and publications, policy engagement
Valente et al. [49]	2007	USA: Massachusetts, Colorado, Adkansas, Iowa & MIssouri	Randomised controlled trial	STEP (Steps Toward Effective Prevention)	Research project	Social network theory	415 (baseline) 406 (follow up) participants 24 coalitions	Self-rated survey	Benchmark achievement, prevention activity progress
Wagner et al. [42]	2009	USA: Colorado	Not specified *(pre-test, post-test)*	Not specified	Unclear	Social capital theory	181 participants 10 coalitions	Self-rated survey	Social capital
Watson-Thompson et al. [51]	2008	USA: Kansas City	Quasi-experimental, interrupted time-series design	Ivanhoe Neighbourhood Council & Northeast Coalition	Grassroots	Institute of Medicine’s Framework for Collaborative Public Health Action in Communities	40 participants 2 coalitions	Self-rated survey	Instances of community and systems change
Watson-Thompson et al. [52]	2014	USA: Mid-west	Between-group randomised controlled trial	Community Anti-Drug Coalitions of America (CADCA)	Unclear	Institute of Medicine’s Framework for Collaborative Public Health Action in Communities	27 participants 10 coalitions	Self-rated survey	Community change (e.g. policy, practice)
Wells et al. [48]	2009	USA: Pennsylvania	Mixed methods	Communities That Care	Unclear	Organisational theory (Hackman)	1,081 (exposure) 1,502 (outcome) participants 45 coalitions	Self-rated survey	Perceived coalition impact
Yang et al. [38]	2012	USA	Analytical cross-sectional	CADCA	Unclear	Socio-ecological Framework & Community Problem Solving and Change Framework	551 participants 551 coalitions	Self-rated survey	Comprehensiveness of strategies, engagement with systems change, facilitating community change
Zeldin et al. [43]	2016	Malaysia	Not specified *(quasi/ pre-test post-test)*	Not applicable	Policy/funding response	Youth-adult partnership	357 (wave 1) 207 (wave 2) participants 3 coalitions	Self-rated survey	Youth empowerment

Author	Outcome measurement tool	SDOH Initiative focus	SDOH measurement tool	Analysis	Analysis type	Analysis level	Mediating effects observed	Quality score (0-8)
Allen et al. [39]	Survey (coalition members)	Family violence	Not evaluated	Hierarchical linear modelling	Reg/Cor Modelling	Individual, coalition	Yes	3
Anderson-Carpenter et al. [46]	Interview (coalition members)	Underage drinking	Not evaluated	Paired-sample t-tests and Two-tailed Person correlations	Sig diff Reg/Cor	Geographical region	Not analysed	6
Brown et al. [28]	Survey (coalition members)	Drug use, violence, crime	Not evaluated	Multiple regression	Reg/Cor	Coalition	Not analysed	6
Calancie et al. [44]	Survey (coalition members)	Food environments	Not evaluated	Structural equation modelling	Modelling	Individual, coalition	Yes	6
Cicognani et al. [29]	Survey (coalition members)	Healthy eating, physical activity, smoking, alcohol consumption, health inequalities	Not evaluated	Regression & SEM	Reg/Cor Modelling	Coalition	Yes	3
Crowley et al. [45]	Survey (coalition members)	Substance abuse	Self report survey (coalition)	Structural equation modelling	Modelling	Not specified	Not analysed	4
Donchin et al. [30]	Survey (coalition members)	Health equity	Not evaluated	Spearman’s correlation & ANOVA	Reg/Cor	Coalition	Not analysed	3
Drach-Zahavy et al. [31]	Interview (coalition members)	Smoking, nutrition, physical activity, health conditions	Not evaluated	Pearson intercorrelations & hierarchical regression analysis	Reg/Cor	Coalition	Not analysed	4
Duran et al. [32]	Survey (coalition members)	Not specified	Not evaluated	Univariate regression	Reg/Cor	Individual	Not analysed	3
Emshoff et al. [47]	Survey (coalition members)	Early years	Self report survey (coalition)	Multi-level modelling	Reg/Cor Modelling	Not specified	Yes	4
Feinberg et al. [33]	Interview (coalition members)	Teen substance use, violence, educational attainment, pregnancy	Not evaluated	Intercorrelations, scatterplots & mediational analysis	Reg/Cor	Coalition	Yes	5
Flewelling et al. [12]	Survey (community)	Underage drinking	Community survey	Mixed model regression	Reg/Cor	Coalition	Not analysed	6
Kegler et al. [34]	Survey (coalition members)	Youth development, civic capacity building, neighbourhood improvement, education (et al.)	Not evaluated	Multi-level mediation analysis	Reg/Cor Modelling	Individual, coalition	Yes	4
Lawless et al. [41]	Survey (coalition members)	Crime, the community, housing and the physical environment, health, education, employment	Community survey	z-scores of a composite index of relative change	Sig diff	Geographic region	Not analysed	2
Mansergh et al. [50]	Survey (coalition members) Document scan	Alcohol and other drug use	Not evaluated	ANCOVA & MANCOVA	Reg/Cor	Individual, coalition	Not analysed	5
Nowell et al. [47]	Survey (coalition members)	Domestic violence	Not evaluated	SEM, ANOVA & OLS multiple linear regression	Reg/Cor Modelling	Individual	Not analysed	5
Oetzel et al. [35]	Survey (coalition members) Interview (coalition members) Document scan Observational data collection	Not specified	Case study & survey	SEM	Modelling	Coalition	Not analysed	5
Powell et al. [36]	Survey (coalition members)	Alcohol, tobacco, and other drug use	Not evaluated	SEM	Modelling	Individual	Yes	5
Ramanadhan et al. [37]	Survey (coalition members)	Health inequities	Not evaluated	Multiple linear regression models	Reg/Cor	Not specified	Not analysed	4
Valente et al. [49]	Survey (coalition members)	Drug prevention	Not evaluated	Confirmatory factor analysis & regression analysis	Reg/Cor	Coalition	Yes	2
Wagner et al. [42]	Survey (coalition members) Interview (coalition members)	Natural resources management	Not evaluated	Multiple regression analysis, general linear model, mediation analysis	Reg/Cor	Individual, coalition, all coalitions	Yes	6
Watson-Thompson et al. [51]	Interview (coalition members) Document scan	Neighbourhood improvement	Not evaluated	Between-group comparison	Sig diff	Coalition	Not analysed	4
Watson-Thompson et al. [52]	Survey (coalition members) Document scan Observational data collection	Substance abuse	Not evaluated	Paired sample t-test	Sig diff	Coalition	Not analysed	4
Wells et al. [48]	Survey (coalition members)	Adolescent risk factors	Not evaluated	Bivariate correlations & regression model	Reg/Cor	Coalition	Not analysed	5
Yang et al. [38]	Survey (coalition members)	Substance abuse	Not evaluated	SEM	Modelling	Not specified	Not analysed	5
Zeldin et al. [43]	Survey (youth)	Youth empowerment, civic engagement	Not evaluated	Other modelling / pathway analysis	Modelling	Not specified	Yes	7

*Quality assessed using the Joanna Briggs Institute Checklist for Analytical Cross Sectional Studies* [27] *(range 0-8)*

*“SDOH” = social determinants of health, “USA” = United States of America, “UK” = United Kingdom, “Reg/Cor” = regression, bivariate correlation or similar analysis, “Sig diff” = significant difference in values between multiple coalitions e.g. an intervention and delayed community, “SEM” – statistical equation modelling, “ANOVA” = analysis of variance, “ANCOVA” = analysis of covariance, “MANCOVA” = multivariate analysis of covariance, “OLS” = ordinary least squares*

### Conceptual framework

Authors cited a variety of guiding frameworks for their research. Five papers cited a collaborative capacity framework, including the Community Coalition Action Theory [34], CSAP Strategic Prevention Framework [13], Institute of Medicine’s Framework for Collaborative Public Health Action in Communities [51, 52], the Food Policy Council Framework [44]. Four used untitled models built through literature reviews [28, 39, 47, 50]. Three studies were grounded in Community Based Participatory Research (CBPR) [32, 35, 37], three in organisational theory [33, 36, 48], and three in a community readiness model [33, 38, 46].

### Intervention target (SDOH)

Five studies included evaluation of the target SDOH [13, 35, 40, 41, 45]. All studies that evaluated the target SDOH featured large, multi-community initiatives that had either regular data collection built into the design [13, 35, 40, 45], or funding specifically allocated to evaluation of the program [41]. Crowley et al.’s research into substance abuse used a self-reported survey of coalition members to measure perceived community behavior change [45], Emshoff et al.’s study addressing health service access evaluated the impact of using service coordination and utilisation data [40], Flewelling et al.’s study focusing on youth alcohol used self-reported survey data from young people [13], Lawless’s multi-focus study addressing worklessness and educational attainment used regional data collected by the social disadvantage research centre [41], and Oetzel et al.’s study, which didn’t specify a health issue, used a community survey to collect data on undefined indicators [35].

### Exposure (collaboration characteristics)

Twenty three studies used participant surveys, most commonly Likert-type questionnaires administered to coalition coordinators or participants [13, 28–30, 34–52]. With the exception of Donchin et al.’s tool [30], which required researchers to allocate a rating to participant responses, all surveys collected self-reported ratings on communication, trust, efficiency, task-focus, decision making and participation. Almost half (*n*=11) of the studies used tools that were tested for reliability, but fewer (*n*=8) were validated. Four studies used participant interviews, with responses scored by researchers on a number of domains to enable quantitative analysis [31–33, 35]. Two studies audited existing coalition documentation, which looked for evidence of coalition characteristics or functioning [35, 46].

### Community outcomes

Community outcome evaluation included proximal (shorter-term) and distal (longer-term) measures (Table 3). Proximal indicators included community capacity or readiness to change [28, 29, 32, 34, 35, 39, 45–47], social capital [34, 39, 42, 44], and member empowerment [29, 32, 36, 39, 43]. Despite similar conceptions of community capacity between studies, a variety of indicators were used. Distal indicators included policy change [30, 37, 52], community change [14, 28, 32, 37, 38, 40, 45, 51, 52], health outcomes [13, 50], and perceived effectiveness [31, 33, 36, 44, 48]. The most common data collection method was a survey [28–30, 32, 34–42, 44, 45, 47–50, 52] or interview [31, 33, 35, 42, 46, 51] of coalition members.

**Table 3:** Summary of community outcome measures

Short-term	Medium-term	Long-term
- Service diversity - Targeting of multiple program and policy sectors - Sustaining the work - Collaborative service delivery - Resource acquisition - Enhanced opportunity for impact - Self-efficacy - Coalition efficacy - Make outcomes matter - Partnership capacity / capability - Synergy - Member capacity	- Community capacity - Social capital - Community empowerment - Awareness (of issue) - Perceived effectiveness	- Community change (not specified) - Health promoting environments - Program, policy and procedure change - Equitable policy change - Health issue specific indicators e.g. prevalence

### Quality appraisal findings

The methodological quality of the included studies varied (Table 2). Twenty studies met >50 percent of the eight quality appraisal standards, and six met ≥75 percent of the standards. Eighteen (69 percent) described participating coalitions and individuals adequately. Validation was the largest quality gap in the appraised studies: eight (31 percent) used validated tools to measure coalition characteristics, seven (27 percent) used validated tools to measure community outcomes, and two (8 percent) used validated tools to measure the target SDOH.

### Data analysis

Most studies (*n*=16) used regression or other analysis of correlation to ascribe links between coalition characteristics and community-level outcomes [13, 20, 28–33, 37, 40, 42, 46–50]. Four of these studies included analysis of mediating factors, to understand how multiple coalition characteristics interact to reinforce or subdue each other’s effects on community outcomes [31, 33, 34, 40]. Ten used a modelling technique, such as structural equation modelling (SEM) [29, 34–36, 38, 39, 43–45, 47]. Of the studies that used mediation analysis or modelling, all found mediational effects relevant to the relationship between coalition characteristics and outcomes. Eleven studies [13, 28–31, 33, 35, 48, 49, 51, 52] analyzed data at a coalition level, three studies [32, 36, 53] analyzed data at an individual respondent level, and five studies [34, 39, 42, 44, 50] included both levels of analysis. Two studies [41, 46] analyzed data at a regional level that included multiple coalitions, and five [37, 38, 40, 43, 45] studies did not specify their unit of analysis.

### Coalition characteristics

There was consistent evidence that coalition characteristics are positively associated with community outcomes (Table 4). There were a range of significant associations between coalition characteristics reported, however these results are outside the scope of this review.

**Table 4:** What works: significant associations between coalition characteristics and outcomes

Domain		Coalition characteristic	Associated outcomes – direct pathways (significance)	Associated outcomes – indirect pathways (intermediary)
Community context	Community resources	Socio-economic position (income, income support, food relief, educational attainment, employment)	Positive: Systems change (shared decision making, *p*<0.05) [47]	
		Community vibrancy (building, youth, housing growth)	Positive: Systems change (coalition finance, *p*<0.05; shared decision making, *p*<0.05) [47]	
		Social capital	Positive: Improved health / health equity (p=0.06) [32], partnership synergy (development of goals and strategies, problem solving, responsive to community needs, teamwork, p=0.05) [32], community transformation (*p*<0.05) [32], institutional change (*p*<0.05) [39], social capital (*p*<0.05) [42]	**Mediation:** Social capital (mediated by success) [42]
	Capacity	Partnership capacity	Positive: Individual member capacity building (p=0.05) [32], community transformation and health equity (*p*<0.05) [32], social capital (member empowerment, relationships, knowledge, credibility, *p*<0.001) [44], perceived impact and synergy (*p*<0.001) [44]	**Pathway:** Perceived effectiveness (via social capital) [44]
		Community psychological, political and financial empowerment	Positive: Number of health promotion changes (*p*<0.05) [31] Negative: Health promoting environments (*p*<0.05) [31]	
		Community readiness to change / capacity	Positive: Community change (*p*=0.066) [46], attitudes and knowledge of prevention (*p*<0.05) [33], intermediate outcome improvement (risk and protective factors, p≤0.05) [45], institutional change (*p*<0.05) [39]	**Mediation:** Perceived effectiveness of coalition’s work (mediated by coalition functioning) [33] **Pathway:** Health outcome and behaviour change (via intermediate outcomes) [45]
Coalition resources	Resource levels	Adequacy of staffing	Positive: Member satisfaction (*p*<0.001) [34]	**Mediation:** Community capacity (new skills, social capital and sense of community, mediated by member satisfaction) [34]
		Resource levels	Positive: Community participation and management (*p*<0.001) [30], knowledge and awareness (*p*<0.01) [47], opportunity and impact (*p*<0.01) [47], social capital (*p*<0.01) [47]	**Barrier:** Barrier to success [33]
		Training and technical assistance	Positive: Coalition outcomes (p value not supplied) [49], ability to establish a vision and mission (*p*<0.05) [52], arrange community mobilisers (*p*<0.05) [52], community readiness to change (p=0.003) [46]	
	Resource management	Community power over resources	Positive: Intermediate community outcomes (*p*<0.01) [35], distal community outcomes (*p*<0.01) [35] partnership synergy (development of goals and strategies, problem solving, responsive to community needs, teamwork, p=0.01) [32]	**Pathway**: Intermediate outcomes (via community involvement in research, positive) [35], **i**ntermediate outcomes (via community involvement in research, positive) [35]
		Joint resource management between partners	Positive: Member agency capacity building (p=0.05) [32], community transformation and health equity (*p*<0.05) [32]	
		Effective management of financial, in-kind and time resources	Positive: Partnership synergy (development of goals and strategies, problem solving, responsive to community needs, teamwork, p=0.001) [32], community transformation and health equity change (p=0.001) [32]	
		Shared resource generation and use	Positive: Program array (*p*<0.05) [47], collaborative service delivery (*p*<0.05) [47]	
Coalition structure	Coordination	Chair tenure	Positive: Collaborative service delivery (*p*<0.05) [47]	
	Age	Coalition age/maturity	Positive: Engagement in systems change (p value not specified) [47], network centralisation (p value not supplied) [37], reciprocity (p value not supplied) [37], number intersectoral connections (p value not supplied) [37], betweenness (p value not supplied) [37]	
	Structure	Formal organisation/structure/agreement	Positive: Health outcome (reduced alcohol use, p=0.039, binge reduced drinking, p=0.031) [12], program array (*p*<0.05) [47], perceived effectiveness (*p*<0.05) [43], social capital (*p*<0.05) [39], equal power between coalition and community (*p*<0.01) [32], community transformation and health equity (*p*<0.05) [32]	**Pathway:** Institutional change (via social capital) [39]
	Size	Coalition size	Positive: Rate of implementation (p value not supplied) [51]	
Member characteristics	Expertise	Health promotion experience of coordinator	Positive: Community participation and intersectoral diversity (*p*<0.05) [30]	
		Experience collaborating	Negative: Trust (*p*<0.05) [42]	
	Diversity	Sectoral diversity (members)	Positive: Number of health promotion actions implemented (*p*<0.05) [31], working on multiple strategies (*p*<0.01) [31], social capital (*p*<0.05) [39] Negative: Member participation (p≤0.001) [34], number health education plans (*p*<0.05) [31]	**Mediation**: Community capacity (new skills, mediated by member participation) [34] **Pathway:** Institutional change (via social capital) [39]
	Empowerment	Psychological and political empowerment	Positive: Perceived effectiveness (*p*<0.01) [36]	
Member engagement and satisfaction	Meetings	Meeting attendance	Positive: Social capital (*p*<0.001) [47], opportunity and impact (*p*<0.05) [47]	
		Proportion of members who spoke in meetings	Positive: Perceived coalition impact (*p*<0.10) [48]	
	Activity	Participation in coalition activities	Positive: Perceived coalition impact (*p*<0.10) [48], barrier to success (p value not supplied) [33], community capacity (p≤0.05) [45]	**Pathway:** Intermediate and health/behavioural outcomes (via community capacity) [45]
		Activity level (meeting frequency and engaging in shared activity)	Positive: Collaborative service delivery (*p*<0.05) [47], finance (*p*<0.05) [47]	
	Duration	Duration of membership	Positive: Knowledge and awareness (*p*<0.01) [47], social capital (*p*<0.05) [47], opportunity and impact (*p*<0.001) [47], and resource acquisition (*p*<0.01) [47]	
		Coalition configuration (extent, duration and focus of member involvement)	Positive: Coalition effectiveness (*p*<0.05) [31] Negative: Number of health plans (*p*<0.05) [31]	
	Satisfaction	Satisfaction with coalition	Positive: Empowerment outcome (leadership competence, *p*<0.05) [43], empowerment outcome (policy control, *p*<0.05) [43]	
Group facilitation	Decision making	Shared decision making	Positive: Community capacity (new skills, p≤0.01) [34], sense of community (p≤0.01) [34], member agency capacity building (*p*<0.05) [32], sustained partnership (*p*<0.05) [32], community transformation and health equity change (p=0.001) [32], empowerment outcome (leadership competence, *p*<0.01) [43]	**Pathway:** Community outcome (school attachment, via program safety) [43], sense of community (via member satisfaction) [34], community capacity (new skills, via member satisfaction and participation) [34]
	Functioning	Internal functioning (resourcing, activity, personal benefits, clear plan, sense of direction)	Positive: Perceived effectiveness (*p*<0.01) [33], attitudes and knowledge of prevention (*p*<0.05) [33]	
		Relationships (leadership, resource management, trust, participatory decision making)	Positive: Intermediate (*p*<0.01) and distal (*p*<0.01) coalition outcomes [35]	
		Task focus	Positive: Community capacity (new skills, p≤0.01) [34]	
		Organisation and resources	Positive: Community participation (*p*<0.01) [30], community management (*p*<0.001) [30]	
		Coalition capacity (development and use of plans, expanded membership)	Positive: Comprehensiveness of strategies (*p*<0.01) [38]	**Mediation:** Community change (mediated by comprehensive strategies) [38]
		Collaboration quality (culture of reflection, interdependence, flexibility, new professional activities)	Positive: Member empowerment (*p*<0.05) [29], sense of community responsibility over the health issue (*p*<0.05) [29], sense of the community contributing to health promotion, *p*<0.05) [29], trust (*p*<0.05) [29], commitment to the work (<0.05) [29], perceived efficacy (*p*<0.05) [29]	**Pathway:** Perceived efficacy (via member empowerment, sense of community responsibility, and sense of the community contributing to health promotion) [29]
	Values	Shared values	Positive: Intermediate (*p*<0.01) and distal (*p*<0.01) coalition outcomes [35], member agency capacity building (*p*<0.05) [32], community transformation and health equity (*p*<0.05) [32] partnership synergy (development of goals and strategies, problem solving, responsive to community needs, teamwork, p=0.05) [32]	**Pathway**: Intermediate and distal community outcomes (via relationship and leadership quality, and synergy) [35]
	Leadership	Leadership quality	Positive: Member satisfaction (p≤0.001) [34], community capacity (new skills, p≤0.001) [34], perceived effectiveness (*p*<0.01) [36], knowledge and awareness (*p*<0.001) [47], social capital (*p*<0.001 [47], *p*<0.01 [39]) opportunity and impact (*p*<0.001) [47], resource acquisition (*p*<0.001) [47], partnership synergy (development of goals and strategies, problem solving, responsive to community needs, teamwork, p=0.001) [32], community transformation and health equity change (p=0.05) [32],	**Pathway:** Perceived effectiveness (via opportunity for leadership roles, psychological empowerment of members, social support between members, and a group based belief system) [36], institutional change (via social capital) [39], community capacity (new skills, via member participation) [34]
	Empowerment	Member empowerment	Positive: Institutional change (*p*<0.01) [39]	
		Members encouraged into leadership roles	Positive: Coalition effectiveness (*p*<0.01) [36]	
	Communication	Communication quality	Positive: Perceived success (*p*<0.05) [42]	
Group dynamics	Conflict	Group cohesion	Positive: Social capital (p≤0.001) [34], sense of community (p≤0.001) [34], perceived coalition effectiveness (*p*<0.01) [36]	**Mediation:** sense of community (via member satisfaction) [34]
		Conflict	Negative: level of implementation (p value not supplied) [33],	**Barrier:** Barrier to implementation [47]
	Support	Supportive relationships	Positive: Perceived effectiveness (*p*<0.01) [36], program safety (*p*<0.001) [43]	**Pathway:** Health outcome (school attachment, via program safety) [43]
		Dialogue and listening (positive attitude, participation and learning from each other)	Positive: Equal power between coalition and community (p=0.05) [32]	
	Trust	Perceived safety, inclusion	Positive: Community connection (*p*<0.01) [43], social capital (*p*<0.05) [39]	**Pathway:** institutional change (via social capital) [39]
		Trust	Positive: Perceived success (<0.05) [42], sustained partnership (*p*<0.05) [32], equal power between coalition and community (*p*<0.05) [32]	
Relationship and network structure	Number	Number of intersectoral partnerships	Positive: Community activity (p≤0.01) [37], policy engagement (p≤0.05) [37], community support (*p*<0.05) [28], sustainability planning (*p*<0.05) [28]	
		Increase in number of social connections	Positive: Community readiness (*p*=0.056) [46], number of community changes (*p*=0.031) [46]	
	Structure	Network density (social network analysis)	Positive: Planning in early stages of coalition (*p*<0.05) [49], Negative: Coalition functioning and progress in later stages of coalition (*p*<0.05) [49]	
		Loosely bound network (part-time and moderate turnover of positions)	Positive: Working on multiple strategies (*p*<0.05) [31], coalition effectiveness (*p*<0.01) [31] Negative: Number of health plans implemented (*p*<0.05) [31]	
		Reciprocity of partnerships	Positive: Community activity (p≤0.01) [37], grant submission (p≤0.01) [37], perceived success (*p*<0.05) [42]	
Community partnership	Community partnerships	Resident involvement	Positive: Community neighbourhood satisfaction (*p*<0.01) [41], perceived neighbourhood improvement (*p*<0.01) [41], feel a part of the community (*p*<0.05) [41], trust the coalition (*p*<0.05) [41], feel they can influence local decisions (p value not specified) [41], individual member capacity building (p=0.03) [32], community transformation and health equity change (p=0.01) [32], equal power between coalition and community (*p*<0.001) [32], intermediate (*p*<0.01) and distal (*p*<0.01) coalition outcomes [35] Negative: Worklessness improvements (p value not specified) [41],	
		Political support	Positive: Equitable policy change (*p*<0.01) [30]	
	Professional partnerships	Links with external entities	Positive: Health outcome improvement (p=0.011) [12]	
		Engagement with health professionals and subject matter experts	Positive: Coalition effectiveness (*p*<0.05) [31], number of health promotion actions implemented (*p*<0.05) [31], healthy physical and social environments (*p*<0.01) [31] Negative: empowerment (*p*<0.05) [31]	
		Participation in community of practice	Positive: Equitable policy implementation (*p*<0.05) [30], degree community participation (*p*<0.01) [30], number intersectoral partnerships (*p*<0.05) [30]	
Planning and implementation	Implementation	Number of actions implemented	Positive: Healthy physical and social environment (*p*<0.05) [31], perceived effectiveness (*p*<0.05) [31]	
		Level of policy implementation	Positive: Increased community capacity (p≤0.05) [45]	**Pathway:** Intermediate and health/behavioural outcomes (via community capacity) [45]
		Collaborative service delivery	Positive: Service diversity (p value not specified) [47]	
		Intervention fidelity	Positive: Perceived coalition impact (*p*<0.05) [48]	
		Partnership synergy (strategic planning, problem solving, teamwork, responsiveness)	Positive: Intermediate (*p*<0.01) and distal (*p*<0.01) intermediate (*p*<0.01) and distal (*p*<0.01) outcomes [35], social capital (*p*<0.05) [42]	
		Governance of the work	Positive: Perceived coalition impact (*p*<0.001) [48]	
	Planning	Have a strategic plan	Positive: Rate of implementation (p value not supplied) [51]	
		Diverse/comprehensive strategies	Positive: Collaborative service delivery (*p*<0.05) [47], community change (*p*<0.01) [38]	
		Number of health plans	Positive: Health promotion actions implemented (*p*<0.01) [31], perceived effectiveness (*p*<0.01) [31]	
		Number of data sources used to inform strategies	Negative: Health outcome (alcohol use, p=0.029) [12]	

Correlations listed are those deemed significant by authors, and that relate to coalition outcomes either directly or indirectly. Non-significant findings have not been recorded in the table. “Community transformation” = health, policy, environmental, financial change, “Number of health promotion actions implemented “ = includes policy change, reform, empowering community, environmental change, and skill development

#### Community context

Nine studies showed significant associations between community context and coalition outcomes [31–33, 39, 40, 42, 44–46]. Socioeconomic position and vibrancy (descriptions in Table 4) were positively associated with systems changes relating to shared decision making (*p*<0.05) [40], coalition resourcing (*p*<0.05) [40], and collaborative service delivery (mediated through chair tenure) [40]. Social capital was positively correlated with medium and long-term community outcomes [32, 39, 42]. Existing capacity, or readiness, within both the community and the coalition was positively associated with a range of short [31], medium [32, 33, 44, 45] and long-term [31, 32, 39, 46] outcomes, though the strength of this relationship weakened after the effect of coalition functioning was controlled for [33].

#### Coalition resources

Nine studies found coalition resourcing to be positively associated with outcomes, including the level of financial resources [30, 47], resource management [35, 40, 49], staffing [34], and training and technical assistance [46, 49, 52]. Resourcing levels were positively associated with community participation (*p*<0.001) [30] and a range of medium-term outcomes such as knowledge and awareness (*p*<0.01) [47] and social capital (*p*<0.01) [47]. There were positive associations between community control over coalition resources and partnership synergy (p=0.01) [32], intermediate outcomes (*p*<0.01) [35], and distal outcomes (*p*<0.01) [35]. Adequate staffing supported community capacity building through increased member satisfaction with the coalition (*p*<0.01) [34], and training and technical assistance supported coalitions through improved short-term [46, 49, 52] and medium-term outcomes [46].

#### Coalition structure

Five studies showed direct, positive associations between formalisation of the coalition (e.g. through a written agreement or formal structure) and coalition outcomes, including health behavior change (*p*=0.031) [13], program array (positive, *p*<0.05) [40], perceived effectiveness (*p*<0.05) [50], social capital (*p*<0.05) [39], equal power between coalition and community (positive, *p*<0.01) [32], community transformation (positive, *p*<0.05) [32], and health equity (positive, *p*<0.05) [32]. Analysis of the relationship between coalition maturity and outcomes showed mixed results. Two studies found significant, positive relationships between coalition age and short-term outcomes, including engagement with systems change (p value not supplied) [47] and community support for the coalition’s work (*p*<0.05) [28]. They also showed improvements in coalition functioning with age, including strategy implementation (p value not supplied) [51], leader-member communication (*p*<0.05) [28] and sectoral diversity (*p*<0.05) [28]. However, multiple studies found that early stages of health promotion focus on needs assessment and planning rather than implementation which may influence results [45, 51], and other studies found no significant relationship between coalition age and longer-term outcomes [42, 48].

#### Member characteristics

Five studies [28, 31, 34, 39, 48] considered the role of sectoral diversity amongst coalition members in driving coalition impact, with three finding significant associations [31, 34, 39]. There was evidence of higher levels of participation in homogenous groups (p≤0.001) [34], and a greater number of actions being successfully implemented (*p*<0.05) [31]. However, looking towards implementation quality, heterogenous groups were more likely to implement diverse strategies that have a systems-change focus (*p*<0.01) [31], and increase social capital (*p*<0.05) [39]. Diverse membership was not directly associated with improved outcomes in three studies [28, 31, 48], was positively associated with coalition outcomes in one study where it was part of a composite measure of coalition capacity (*p*<0.01) [35], and was negatively correlated with community capacity in one study (p≤0.001), unless there was a high level of member satisfaction, which mediated the result (p≤0.001) [34]. In this context, satisfaction was shaped by shared decision making, task focus, frequency and productivity of communication, group cohesion, quality leadership, and adequate staffing. The psychological and political empowerment of members was positively associated with perceived effectiveness (*p*<0.01) [36], while past experience influenced collaborative practice. Coalitions led by coordinators with a health promotion background were more likely to see community participation and intersectoral diversity (*p*<0.05) [30], while a history of collaborative work was negatively associated with trust (*p*<0.05) [42]. The number of years working in the field and educational attainment of coalition members did not show significant associations with community outcomes [47, 48].

#### Member engagement and satisfaction

Coalition member engagement was predominately measured through time and participation, both in meetings and other coalition activities. Member engagement was positively correlated with community outcomes in eight studies [31, 33, 34, 40, 43, 45, 47, 48]. Greater engagement was also associated with better coalition management (*p*<0.001) [30], more collaborative service delivery (*p*<0.01) [40], increased member and partner organisation capacity [43, 47], and coalition finances (*p*<0.05) [40]. More specifically, coalition attendance [47] and the time spent dedicated to the coalition beyond meetings [33, 40, 45, 48] were correlated with perceived coalition impact, however the amount of time spent in meetings, and talking in meetings, were not [48]. One study found that member participation and satisfaction mediated relationships between other coalition characteristics such as sectoral diversity, decision making, cohesion, leadership, and staffing, and community outcomes [34]. Member empowerment, the extent to which coalition members were encouraged to step into coalition leadership roles, and sense of connectedness and cohesion, predicted coalition effectiveness [39, 44].

#### Coalition facilitation and communication

Twelve studies showed associations between the stability and quality of coalition leadership and community outcomes [29, 30, 32–36, 38, 39, 42, 43, 47]. Five studies found a direct, positive correlation between higher quality coalition leadership and community outcomes, including community capacity (p≤0.001) [34], perceived effectiveness (positive, *p*<0.01) [36], social capital (*p*<0.001 [47], *p*<0.05 [39]), and community transformation and health equity change (positive, p=0.05) [32]. Nowell and Foster-Fishman [47] found that member perception of leadership and decision making within a coalition was positively correlated with coalition functioning including gains in knowledge and awareness (*p*<0.01), opportunity and impact (*p*<0.001), and resource acquisition (*p*<0.01).

Collaborative capacity or functioning was positively associated with partnership synergy (working well together), community readiness or capacity to change, social capital, project efficacy, and intermediate and distal community outcomes [28, 29, 34–36, 39, 42, 44]. Studies that investigated discreet qualities, demonstrated significant associations between coalition effectiveness and open and cohesive group dynamics [34, 36, 39, 44], leadership [39, 44], supportive and trusting relationships [36, 42], communication quality [28, 42], internal organisation and structure [13, 31, 33, 39, 44], decision making [34], and task focus [34]. Partnership structural values, which was a composite construct that included bridging social capital and shared values, was associated with improved intermediate and distal community outcomes (*p*<0.01) [35].

#### Group dynamics

There were positive correlations between community outcomes and cohesion, support, dialogue, trust, and group safety [34, 35, 43]. Mutual support and dialogue showed associations with perceived effectiveness (*p*<0.01) [36], group safety (*p*<0.001) [43], and equitable power dynamics between the coalition and wider community (p=0.05) [32]. One study concluded that member turnover and conflict were important factors when assessing internal functioning, finding that coalitions with the lowest level of implementation reported higher levels of infighting (p value not supplied) [33]. In a youth-adult partnership context, youth voice was positively associated with the target community outcomes of youth leadership (*p*<0.01), policy control (*p*<0.001), and perceived program safety (*p*<0.001) [43].

#### Relationship and network structure

Three studies found significant, positive correlations between the number of collaborative partnerships and community outcomes [28, 37, 46]. An increase in collaborative partnerships over time was associated with the number of community changes achieved (p=0.31) [46] and community readiness to change (p=0.056) [46]. Social network analysis (SNA) showed a significant relationship between intersectoral out-degree, or the number of intersectoral relationships reported by coalition members, and level of community activity (p≤0.01) [37] and policy engagement (p≤0.05) [37]. There was also a correlation between the percentage of intersectoral ties that were reciprocal (i.e. both parties said it was important) and level of community activity (p≤0.01) [37], grant submissions(p≤0.01) [37], and perceived success (*p*<0.05) [42]. Two studies investigating network density had opposing findings. Drach-Zahavy et al. [31] found that a loosely bound network, emphasising part-time and moderate turnover of positions, was positively associated with working on multiple strategies (*p*<0.05) and coalition effectiveness (*p*<0.01). A tightly bound coalition network was positively associated with the number of health plans implemented (*p*<0.05) [31]. Conversely, Valente et al [49] found that network density, defined as the total number of ties divided by the total number of possible ties, was positively associated with coalition planning near its inception (*p*<0.05), but significantly, inversely correlated with coalition functioning (*p*<0.05) and planning (*p*<0.05) at 18 months.

#### Community partnership

Seven studies investigated associations between engagement with community members [32, 35, 41] or professionals [13, 30, 31, 33] external to the coalition, and coalition outcomes. Community engagement was positively related to community empowerment in two studies [32, 41], coalition outcomes in three studies [32, 35, 41], and negatively associated with at least one target health outcome in two studies [31, 41]. Maintaining professional partnerships was positively associated with working on multiple strategies (*p*<0.05) [31], coalition effectiveness (*p*<0.05) [31], healthy environments (*p*<0.01) [31]. Political support was found to be strongly and positively associated with equitable policy change (*p*<0.01) [30] and engaging with communities of practice (p=0.043) [30], which itself was supportive of a range of short-term outcomes [30]. One study did not find significant associations between community partnerships and coalition outcomes, but concluded that external linkages may be more important for coalitions that rely on local organisations to provide resources for the work [33].

#### Planning and implementation

Positive associations were observed across ten studies between the use of best practice [4] health promotion planning and evaluation, and coalition outcomes [13, 31, 33, 35, 38, 40, 42, 45, 48, 51]. The existence of strategic plans was positively associated with number of strategies (*p*<0.05) [31], number of strategies implemented (p value not supplied) [51], and coalition effectiveness, health promoting environments, and community empowerment (in a pathway via the number of strategies, *p*<0.05) [31]. The number of data sources used to inform strategic planning (p=0.029) [13], comprehensiveness of the strategies (*p*<0.01 [38], *p*<0.05 [40]), board governance of the coalition’s activities (*p*<0.001) [48], and implementation fidelity (*p*<0.05) [48] were all associated with coalition impact. The level of policy change (p≤0.05) [45] and number of programs implemented (*p*<0.05) [31] were positively correlated with community change outcomes. Coalitions were more likely to adhere to best practice health promotion, and to produce community outcomes when they developed their operational and problem-solving capacities, such as through training [38, 49, 52].

## Discussion

### Key themes

#### Research methods

Our review found few studies (*n*=26) globally, over the past 40 years, that analyzed the relationship between coalition characteristics and outcomes in health promotion initiatives that targets the SDOH. Studies had a limited geographic spread and were published recently, with over half (*n*=15) the studies being published in the past decade. There was no unifying theory guiding the research, which possibly drove the heterogeneity of study designs, measures, and analyses. Due to the cross-sectional research design used in many studies included in this review, it was difficult to assign directionality to results. For example, it is unclear if coalitions and communities who experience positive impacts are more likely to rate strategic planning as important, or if coalitions with stronger strategic planning deliver better outcomes [51]. The same can be said for the relationship between resource acquisition and knowledge, impact, and social capital [47]. Future research should use more rigorous and consistent methods, and longer time scales, in order to better understand the impact of interventions to improve coalitions.

#### Definition and measurement of outcomes

The measurement of coalition characteristics and outcomes varied greatly, with the majority of studies using unvalidated, self-reported measures of perceived functioning and/or effectiveness. Several indicators were classified inconsistently between studies as process, impact, or outcome measures. For example, community empowerment appears as a coalition characteristic in some studies [31, 35, 41] and outcome in others [29, 36, 43]. The variation in indicators used to measure similar constructs made it difficult to draw conclusions on ideal measures of coalition functioning and their impacts on community outcomes, as some were shown to be more relevant than others. For example, meeting attendance was commonly used as a measure of coalition engagement, but studies did not capture information about the quality and purpose of engagement, which is likely to be most relevant to coalition effectiveness [48]. This was magnified where researchers used composite constructs, combining several indicators to measure coalition effectiveness [35]. The majority of studies did not include distal outcome evaluation relating to their target SDOH, relying instead on self-reported indicators of shorter-term organisational, attitudinal, policy, systems or environmental change. In their review of evaluation methods used in coalitions, Kegler, Halpin and Butterfoss [6] note that large, government-funded initiatives often provide communities with a list of acceptable activities, based on established evidence. If a relationship between particular interventions and outcomes has already been established, coalitions might focus their evaluation resources on shorter-term goals, rather than replicating existing research. This may explain the outcome reporting gaps in the studies included in this review. Further, authors used inconsistent cut-off points to determine significance, possibly driven by sample size, data collection tools, and the types of associations investigated. However, a greater focus on evaluating outcomes using validated, objective tools is required to reduce the risk of bias. Evidence of mediational effects and complex relationships between variables in studies that used SEM suggest that this approach to developing a framework for understanding coalitions might be more useful than more traditional, linear models of cause and effect. For example, Kegler and Swan [34] showed that the relationship between coalition characteristics and community capacity was mediated by the level of participant satisfaction, and the model by Oetzel et al. [35] showed that some characteristics were better predictors of success than others. Understanding the relative importance of various coalition characteristics, and the way they enhance or suppress other determinants of success, offers practitioners the chance to direct their efforts to aspects of coalition functioning that give the best return on investment.

#### Best practice health promotion

Coalition researchers who favour a collectivist approach have defined new concepts and language to describe why coalitions work well, such as synergy and emergence, to reflect the view that a key driver of coalition success had not been captured in existing health promotion frameworks [21, 54]. However, this review showed that well-established, best-practice health promotion approaches are likely key determinants of coalition outcomes: well informed, multi-pronged strategies that were implemented, monitored, and included provisions for building capacity in the people leading them as well as the wider community, were associated with coalition success [13, 31, 38, 48, 49, 51, 52]. While the consistency of evidence that health promotion best practice is critical to success indicates that coalition characteristics will not fix a problem or create change on their own [45], it is likely that aspects of coalition structure and function, in particular group facilitation, have an important role in influencing health promotion outcomes [29, 30, 32–36, 38, 39, 42, 43, 47].

#### Facilitation, leadership and power sharing

The findings of this review that facilitation and leadership are critical to success, are consistent with earlier reviews. Costumato [55] found that power sharing, trust, leadership style and formalisation can increase the effectiveness of public interagency collaboration. Brush et al. [56] found that member diversity, power sharing, decision making, engagement, trust, conflict resolution, fair allocation of resources, and moving research into systems and policy change are critical success factors in community research partnerships. Hoekstra et al. [57] found that power dynamics between partners, including co-production of knowledge, meaningful stakeholder engagement, building capacity and resources, and considering ethical issues are important in research partnerships. An interesting finding of this review is the importance of health promotion skills in the coalition coordinator, due to their ability to support diversity and community participation [30]. In their critical review of Collective Impact initiatives, Ennis and Tofa [5] note that the complexity of coalition models, and importance of addressing power and equity in the work, requires skill and attention. In this context, health promotion professionals may contribute as much through partnership brokerage and equity planning as they do through technical skills such as strategic planning.

#### Diversity and conflict

Coalition membership may improve effectiveness through the capacity it builds in members and member organisations, including awareness, social capital, enhanced opportunity and impact, and resource acquisition [47]. Whether member diversity had a positive impact on outcomes depended on the aims of the coalition, and the mechanisms put in place to assure harmony [31, 34, 48]. Membership diversity appeared to be a high-risk, high-reward proposition. Diversity, and the looser relationships that can result, were important in coalitions where multi-strategy systems change was the goal, as long as high quality leadership and good conflict resolution was in place [31, 34, 48]. If these elements were not a focus of coalition functioning, there was evidence that members would engage less in both current and future coalition work [42]. Homogeneity and closer relationships tended to result in greater participation, and faster, less complex implementation and results [31, 39]. Where a quick start or relatively simple solution is needed, beginning the work in a high-trust, familiar group may be beneficial. Prior assertions on the role of diversity in coalitions have been largely theory driven [58], or devoid of nuance about when, why, or how diversity might influence outcomes [16]. Studies that focus on business team performance explore possible mechanisms behind reduced outputs in diverse groups such as increased conflict, the challenge in integrating practices, values, and activities, a need for formalisation to facilitate centralised decision making, lower starting levels of familiarity and communication, which are necessary for problem solving in collaborative work, and lower participation from people when they perceive that they are different to the rest of the group [59–61]. Studies in the same field also demonstrate that diverse groups develop more creative solutions [62]. On the whole, the impact of diversity in business teams is consistent with the findings of this review, and indicate that more research into how to overcome challenges associated with diversity is required, as the outcomes are worthwhile. This need for relationship building and working through conflict should be considered when developing timelines and funding arrangements for coalitions: unless a coalition has existed for some time already, there may need to be significant time and resourcing dedicated in the early stages, to ensure that decision making and conflict management processes support effective practice later on.

#### Community engagement

Community member involvement was, unexpectedly, negatively correlated with outcomes in two studies [31, 41]. Given that diversity in coalition membership can lead to outcomes taking longer to emerge, and that how well the group is managed has a strong influence on this, the time scale of the studies that evaluated coalitions including community members may have been too short [31, 33, 41]. Another possibility is the relative power of citizens in effecting systems change is low, when compared to government, universities, and other institutions that commonly partner in coalitions [63]. The influence of starting socio-economic position and social capital on the likely success of coalitions shows that coalitions are really only effective if they, or the members, have power through access to resourcing, decision making, and political influence [32, 39, 40, 42]. A group of thoughtful, committed citizens might be able to change the world, but only where they have access to the tools and resources to do so.

### Strengths

This systematic review was the first to provide a systematic, rigorous exploration of empirical research on the relationship between coalition characteristics and community outcomes globally, using a comprehensive search of six databases. Where much prior research on this topic utilises reflective analysis [16, 17], the research question and inclusion criteria of this review ensured that all included studies featured a quantitative analysis of the influence of coalition characteristics on community outcomes. This offers an opportunity to assess the strength of quantified relationships, rather than repeating existing theory on the topic of collaboration. This review included a range of interventions using systems theory, which has been absent in previous reviews [6]. The diversity of theoretical frameworks and settings in the included studies improves generalisability of results, as other notable reviews focus on one particular methodology such as CBPR [56], or setting such as the public service [55] or research [57].

#### Limitations

There are several limitations to this review, including that there was only a small number of homogenous studies that met the inclusion criteria, precluding a meta-analysis. As with all systematic reviews that only include published literature, the evidence synthesis could also be limited by publication bias, where studies with neutral or negative results may not be published, thus skewing results. Only English-language studies were included, excluding research reported in other languages [64]. The review excluded 174 qualitative studies showing that most research published on the topic of community-based coalition is qualitative. The focus of the review was on measures of community coalition functioning, and while the qualitative studies provide rich detail, they do not provide insight on how these things are measured quantitatively.

#### Implications for policy and practice

The findings of this review direct practitioners to invest their energy in coalition characteristics to produce success, and researchers to guide future research to validate theoretical frameworks of coalition functioning. Coalition practitioners would benefit from using coalition models to enhance best-practice health promotion approaches, rather than replace them. Issues of power sharing, conflict management, and collaborative leadership should be active considerations in the design and implementation of coalition work, with more traditional planning and evaluation staying at the centre of the approach. Future research should focus on evaluating community outcomes, rather than perceived effectiveness or other shorter-term measures of success. Coalition characteristics and outcomes should be evaluated using validated tools, to strengthen the quality of research in this field. Study designs that allow for multiple data collection points and a quantitative analysis of change over time is needed to understand causation in efforts to improve coalition performance and outcomes. Due to the complex and non-linear relationships between coalition characteristics and community outcomes, analytical methods addressing this complexity such as SEM are best placed to inform future theoretical frameworks and evaluation.

## Conclusion

Despite the wider recognition of the importance of coalitions in health promotion work, our study found a paucity of literature, with high heterogeneity between the small number of studies published over the past four decades. Existing literature demonstrates that coalition characteristics, alongside best practice health promotion planning and evaluation, influence community outcomes. Statistically significant associations were noted between community outcomes and wide range of coalition characteristics, including community context, resourcing, coalition structure, member characteristics, engagement, satisfaction, group facilitation, communication, group dynamics, relationships, community partnership, and health promotion planning and implementation. Further research using consistent description and measurement of coalition characteristics and outcomes, empirical and validated evaluation measures, and analytical methods that consider the interrelationship of variables such as SEM, is warranted.

## Supplementary Information

**Additional file 1.** Search strings. A full list of search terms used to conduct the systematic review.

**Additional file 2.** Data extraction table. A copy of the table used to extract data from studies included in the systematic review.

## Data Availability

The datasets analyzed as part of this review are available from the corresponding author on reasonable request.

## Abbreviations

ANCOVA: analysis of covariance
ANOVA: analysis of variance
CADCA: Community Anti-Drug Coalitions of America
CBPR: community based participatory research
CSAP: Centre for Substance Abuse Prevention
FPC: food policy council(s)
FVCC: Family Violence Coordinating Councils
MANCOVA: multivariate analysis of covariance
OLS: ordinary least squares
SDOH: social determinants of health
SEM: structural equation modelling
SPF-SIG: Strategic Prevention Framework State Incentive Grant
STEP: Steps Towards Effective Prevention
UK: United Kingdom
USA: United States of America
WHOSTOPS: Whole of Systems Trial of Prevention Strategies for Childhood Obesity
