# Supplementary material for: The impact of coalition characteristics on outcomes in community-based initiatives targeting the social determinants of health: a systematic review
Source: BMC Public Health. 2022 Jul 15;22:1358. doi: 10.1186/s12889-022-13678-9 (PMC9288063; doi:10.1186/s12889-022-13678-9)
Supplement: Supplementary file 1 — Additional file 1. Search strings. A full list of search terms used to conduct the systematic review. [file 12889_2022_13678_MOESM1_ESM.docx]

# Additional File 1: Search strings

| Key concept | Search string | Field |
| --- | --- | --- |
| Collaboration | committee %2 (steering OR working OR advisory OR action) OR group %2 (steering OR working OR advisory OR action) OR joint %2 (work or working or initiative) OR “task force” OR network OR collective OR cooperative OR partnership OR alliance* OR coalition OR collaborat* | Title, Abstract |
| Community-based initiatives | "community-based" OR "community based" OR community %2 (led OR driven OR advisory OR coalition OR network OR involvement OR “academic partnership” OR participation OR empowerment) OR participatory OR "community health planning" | Title, Abstract |
| Prevention of health and social issues | prevent* OR change* OR outcome* OR address* OR health* OR social issue* or social problem* OR “social determinant” OR ableism OR aboriginal OR addiction OR “asylum seeker” OR bisexual OR CALD or “culturally and linguistically diverse” OR cultural diversity OR climate OR disability OR discrimination OR “early years” OR education OR “educational attainment” OR employment OR environment* OR food %2 (access OR desert OR insecurity OR security) OR gay OR gender OR gender %2 (diverse OR diversity) OR health services OR income OR “income inequality” OR indigenous OR intersex OR LGBT* OR lesbian OR marginalised OR marginalized OR minority OR obes* OR oppress* OR poverty OR queer OR race OR racism OR racist OR refugee OR sexism OR sexuality OR “sexual diversity” OR “sexual orientation” OR social %2 (capital OR exclusion OR gradient OR “safety network” OR support) OR stress OR trans OR transgender OR “transport access” OR transportation OR unemployment OR violence OR work OR “working conditions” | Title, Abstract |
| Evaluation | evaluat* OR dynamic* OR function* OR character* OR measure* OR assess* OR apprais* OR correlat* OR causal link OR causation OR causality OR causative OR factor OR factors OR monitoring OR learning OR “community based participatory research” OR “action research” OR explor* OR tool OR toolkit OR framework OR synergy OR closeness OR effectiveness OR “information sharing” OR engagement OR structure OR function OR functionality OR functioning OR success OR failure OR trust OR leadership OR relationships OR output OR outcome OR impact OR procedure* OR resource OR resourcing OR resources OR communication OR "network factors" | Title, Abstract |

Boolean search terms legend: * = root word, or that phrase plus any ending, and %2 = ‘near 2’, or within two words of each other
