# Supplementary material for: The impact of coalition characteristics on outcomes in community-based initiatives targeting the social determinants of health: a systematic review
Source: BMC Public Health. 2022 Jul 15;22:1358. doi: 10.1186/s12889-022-13678-9 (PMC9288063; doi:10.1186/s12889-022-13678-9)
Supplement: Supplementary file 2 — Additional file 2. Data extraction table. A copy of the table used to extract data from studies included in the systematic review. [file 12889_2022_13678_MOESM2_ESM.docx]

# Additional File 2: Data extraction table

| **Item** | **Example question** |
| --- | --- |
| Study title | What is the title of the study? |
| Author/s | Who are the authors? |
| Year/s | When was the study published? |
| Location/s | Where were the coalitions based? |
| Coalition name | What was the name of the coalition? |
| Focus/es | What were the social determinants of health targeted by the coalition?  Who was the target population? |
| Theoretical framework | What was the conceptual or theoretical framework that underpinned the coalition? |
| Coalition members | Who was part of the coalition?  Was the target / wider community involved? |
| Resourcing | What resources were available to the coalition?  Was coordination of the coalition specifically resourced? How? |
| Logic model | Was a logic model used to guide the work of the coalition? |
| Formation | Was there a history of coalitions or networks?  How and why did the coalition start? |
| Researcher involvement | Were researchers involved with the coalition, for example during implementation? If yes, when and how? |
| Study design | What was the study design? |
| Participants | Who and how many participated in the study (both individuals and coalitions)? |
| Condition | Was change in the target SDOH measured? If yes, how? |
| Exposure | Which coalition characteristics were measured, and how?  What indicators were used?  How was data collected? |
| Outcome | How was coalition impact measured?  What indicators were used?  How was data collected? |
| Analysis | How did the study attribute the change/s to the collaboration? (e.g. tool/method) |
| Mediational effect | In pathway or statistical modelling studies, were mediating effects observed between any variables?  If yes, what were they? |
| Findings | Was there evidence of a link between coalition characteristics and outcomes? If yes, what were the findings? |
| Strengths | What are the strengths of the study? |
| Weaknesses | What are the weaknesses of the study? |
| Quality | What was the quality score of the study (using JBI tool)? |
